# Local Order in Liquid Gallium–Indium Alloys

**DOI:** 10.1021/acs.jpcc.3c03857

**Published:** 2023-08-09

**Authors:** Alfred Amon, Philip A. Chater, Gavin Vaughan, Rachael Smith, Christoph G. Salzmann

**Affiliations:** † Department of Chemistry, University College London, 20 Gordon Street, London WC1H 0AJ, U.K.; ‡ Diamond Light Source Ltd., Harwell Science and Innovation Campus, Didcot OX11 0DE, U.K.; § European Synchrotron Radiation Facility, BP-220, Grenoble Cedex 9 F-38043, France; ∥ Material Science Division, Lawrence Livermore National Laboratory, Livermore California, 94550, United States

## Abstract

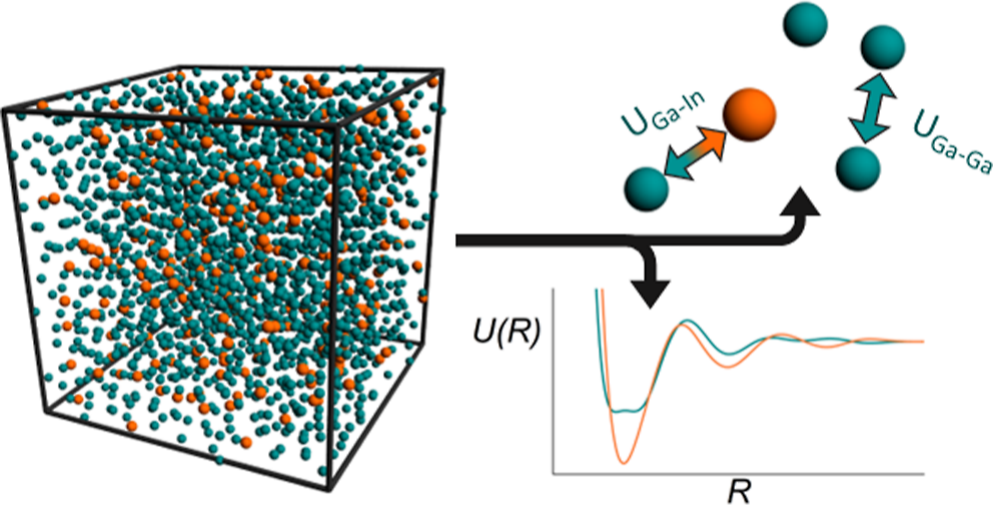

Liquid metals such as eutectic Ga–In alloys have low melting points and low toxicity and are used in catalysis and micro-robotics. This study investigates the local atomic structure of liquid gallium-indium alloys by a combination of density measurements, diffraction data, and Monte-Carlo simulation via the empirical potential structure refinement approach. A high-*Q* shoulder observed in liquid Ga is related to structural rearrangements in the second coordination shell. Structure analysis found coordination environments close to a random distribution for eutectic Ga–In alloy, while electronic effects appear to dominate the mixing enthalpy.

## Introduction

Sitting at the border between transition-metal and metalloid elements, gallium displays one of the most puzzling structural chemistries among the elements. The complex crystal structure of its room temperature stable modification α-Ga features gallium dimers with mixed covalent and metallic bonding character.^1^ Here, every atom is coordinated by one neighbor at a covalent bonding distance of *d*_Ga–Ga_ = 2.48 Å and six neighbors at a larger distance (>2.7 Å). Also, the metastable and high-pressure allotropes form structure types uncommon for metallic elements.^2^ Due to its non-close-packed crystal structure, α-Ga experiences a 3% density increase upon melting, with a concomitant increase of coordination number (CN) from 1 + 6 to about 11.^3,4^ Speculations about the persistence of Ga_2_ pairs^5^ or larger clusters^6,7^ upon melting of the α-Ga modification initiated investigations of the liquid structure in an attempt to explain the characteristic high-*Q* shoulder observed on the first diffraction peak.^8,9^ This shoulder is even more pronounced in the supercooled liquid^10^ and resolved as a separate peak in amorphous gallium films (CN = 9.3).^11^ Besides covalent Ga–Ga bonds in the first coordination shell, rearrangements in the second shell, due to the influence of medium-range Friedel oscillations, have been held responsible for the structural anomalies.^9^ Its anomalous properties sustain ongoing interest to understand the structure of liquid gallium.

Room-temperature liquid alloys, also known as liquid metals, typically have liquidus temperatures below 300 °C and are based on the low-melting elements gallium, bismuth, indium, cesium, sodium, and mercury as majority components.^12^ More recently, liquid metals were discovered as heterogeneous catalysts with the renewable surface for CO_2_ reduction^13,14^ or petrol refining,^15^ templates for synthesis of 2D-materials,^16^ for tailored growth of microcrystals^17^ and as electrode material in all-liquid batteries.^18,19^ In particular, gallium alloys display low-melting points, wide liquid range, low toxicity, and chemical inertness making them suitable as liquid-metal inks for flexible printed electronics^20,21^ or as pumps and actuators in microfluidic devices.^22−25^ A suspension of magnetic particles in liquid gallium can function as a shape-shifting miniature machine with field-assisted solid–liquid transition.^26^

Like the pure element, hypoeutectic gallium alloys display strong undercooling^27^ and in particular the low melting binary eutectic Ga_0.858_In_0.142_ (liquidus temperature *T*_liq_ = 15 °C) and the ternary eutectic Ga_78.3_In_14.9_Sn_6.8_ (*T*_liq_ = 13.2 °C), known as “Galinstan” are of technological interest.^12^ Early X-ray scattering studies of liquid Ga–In alloys found a rapid increase of the nearest-neighbor interatomic distance in the pair correlation function with rising indium content, which flattens out above 50 at. % In and a maximum of the first shell coordination number (CN_avg_ ≈ 12.3) around the equiatomic composition, showing little temperature dependence.^28^ Ding et al. postulated the appearance of clusters reminiscent of crystalline structures for alloys greater 30 at. % In.^29^ The pressure-induced crystallization of liquid Ga_86_In_14_ above 3.4 GPa was studied by X-ray diffraction and molecular dynamic simulations.^30,31^ Around 400 K, a discontinuous change in coordination number was observed in the binary eutectic composition and interpreted as a rearrangement in the first and second coordination shell.^32^ Zhao and co-workers investigated the melt fragility of supercooled Ga–In melts by X-ray absorption spectroscopy, diffraction, and viscosimetry, observing distinct changes in the viscosity below the liquidus temperature and suggesting the presence of low-coordinated polyhedra in the supercooled liquid.^33,34^ A combination of synchrotron X-ray diffraction and ab-initio molecular dynamics simulations, however, observed no abnormal structural changes upon supercooling of Ga–In eutectic alloy.^31^

**Figure 1:**
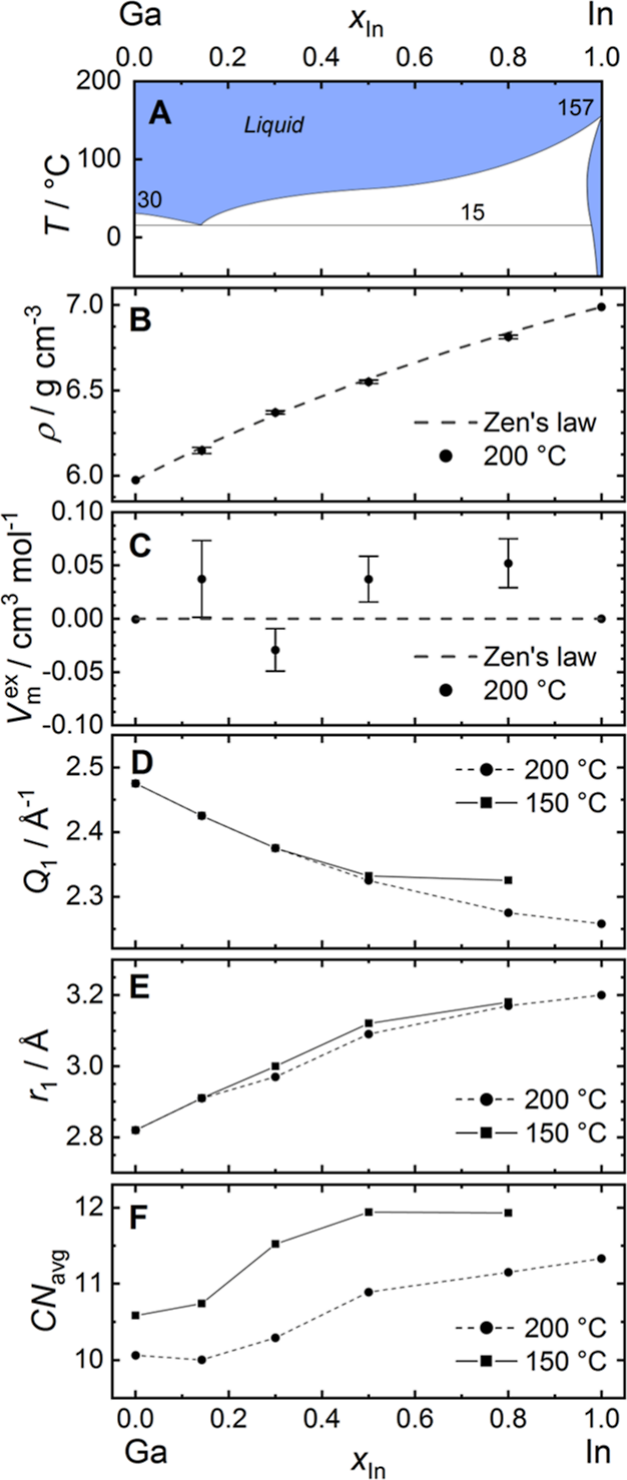
Selected physical properties of the Ga–In system. (A) Reported phase diagram reproduced with data from ref (35). Numbers in the figure indicate the melting points of pure elements and the eutectic temperature. Experimental values for (B) volumetric mass density ρ, and (C) derived molar excess volumes *V*_m_^ex^ at *T* = 200 °C. (D) Position of the first maximum *Q*_1_ in the total structure factor *S*(*Q*) and (E) position of the first maximum *r*_1_ in *G*(*r*). (F) Average coordination number CN_avg_ in the first coordination shell. Lines in (D–F) are guides to the eye. Error bars indicate one e.s.d.

Experimental phase diagram studies and thermodynamic measurements in liquid Ga–In system indicate near regular solution behavior.^35−44^ The parabolic trend of the mixing enthalpy with a maximum of +1.1 kJ mol^–1^ at the equiatomic composition indicates preferred homoatomic interactions in the liquid.^41^ Having a metallic radius difference around 18%, alloys of the isoelectronic elements gallium and indium are just above the Hume-Rothery solubility criterion,^45^ as evidenced by complete miscibility in the liquid phase but asymmetric miscibility in the solid (Figure 1A). Around 50 at. % In, the Ga–In phase diagram (Figure 1A) features a flattened liquidus slope, which can be related to the positive mixing enthalpy in the liquid phase.^46,47^

In this study, we have collected density measurements, synchrotron, and neutron diffraction data combined with Monte-Carlo-based empirical potential structure refinements (EPSR) to study the structure of liquid Ga–In alloys. In particular, we aim to understand (i) the origin of the high-*Q* shoulder on the first diffraction peak of liquid gallium; (ii) whether liquid Ga–In alloys can be treated as ideal solutions; and (iii) if are there preferred atomic interactions leading to the suggested demixing tendencies.

## Methods

Sample handling was carried out in a glovebox system with a nitrogen atmosphere (*p*O_2_ < 0.5 ppm). Liquid alloy samples were prepared by combining stoichiometric amounts of gallium (Acros, 99.99%) and indium (Aldrich, 99.99%) in glass vials and heating for 1 h to 200 °C to ensure homogenization.

### Density Measurements

Density measurements were performed in a borosilicate glass pycnometer after Guy-Lussac (approx. 1 cm^3^ inner volume). The pycnometer was calibrated at 200 °C with pure gallium (reported density of ρ(200 °C) = 5.9737(18) g cm^–3^^48^). Density data are therefore given with respect to this value. The pycnometer (flask and capillary plug) was weighed at room temperature in an empty state, filled with about 0.8 cm^3^ of liquid alloy sample, and weighed. The residual volume was filled with degassed silicone oil (oil bath grade). The filled pycnometer flask and capillary plug were heated and equilibrated at *T* = 200 °C ± 3 °C. The capillary plug was inserted at 200 °C and the expelled excess silicone oil was removed. Then, the cooled-down pycnometer was weighed again to correct the contribution of the silicon oil. The volumetric mass density is reported as the mean value and estimated standard deviation from three independent measurements per sample composition.

### X-ray Diffraction

X-ray diffraction experiments for temperatures of 150 °C and below were conducted at the ID15A beamline of the ESRF (Grenoble, France) using a wavelength λ = 0.125234(1) Å with a Dectris Pilatus3X-CdTe-2M detector.^70^ X-ray diffraction experiments at 200 °C were conducted at the I15-1 beamline of the Diamond Light source (Rutherford-Appleton Laboratory, Didcot, United Kingdom) λ = 0.16167(1) Å. Liquid alloy samples were contained in fused silica capillaries and heated using a hot gas blower. Data reduction, normalization, and correction for furnace and capillary background contributions, beam polarization, attenuation effects, and multiple scattering were done using the DAWN^71^ and GudrunX^72^ software packages.

### Time-of-Flight Neutron Diffraction

Time-of-flight neutron diffraction data were collected on the GEM beamline of the ISIS spallation source (Rutherford-Appleton Laboratory, Didcot, UK). Samples were measured in fused silica ampoules of 10 mm outer diameter and 1 mm wall thickness. Alloys with higher indium content were not accessible to neutron diffraction, due to the strong neutron absorption by indium. Only the time-of-flight data below a neutron energy of 0.33 eV were used to avoid the strong neutron resonance of indium.^73^ Further details on data collection and correction can be found in ref (74). For the EPSR analysis of liquid Ga and Ga_0.858_In_0.142_, simulation boxes containing 1000 Ga atoms, and 1716 Ga plus 284 In atoms, respectively, were constructed according to experimental densities. After structure randomization, the structure model was refined to energy convergence employing only set Lennard-Jones potentials. The Lennard-Jones distance parameters σ were selected according to atomic radii and then iterated to get the best possible agreement with experimental data. Then, the maximum amplitude for the empirical potentials was increased in steps of 0.5 kJ mol^–1^ until no further improvement of the model was achievable. For the final analysis, 3000 or more simulation snapshots were accumulated and the ensemble was averaged.

## Results and Discussion

The volumetric mass density ρ of the liquid Ga_1–*x*_In_*x*_ alloys (*x*_In_ = 0.0, 0.142, 0.3, 0.5, 0.8, 1.0) at *T* = 200 °C ranges from ρ = 5.974(2) g cm^–3^^48^ for pure gallium to ρ = 6.99(1) g cm^–3^ for pure indium and exhibits a nonlinear change with composition (Figure 1B, Table S1). Zen’s law predicts a linear change in molar volume for ideal solutions, corresponding to constant partial molar volumes, which results in a nonlinear function for the volumetric mass density.^49^ The slight positive deviation from linear behavior of the experimental density values is in excellent agreement with the theoretical density (dashed line in Figure 1B) corresponding to linear behavior of the composition averaged molar volume. The derived values for the molar excess volumes *V*_m_^ex^ show no significant deviation from ideality within experimental error (Figure 1C) and did not show the irregular behavior observed by Ben Shalom et al.^39^

**Figure 2:**
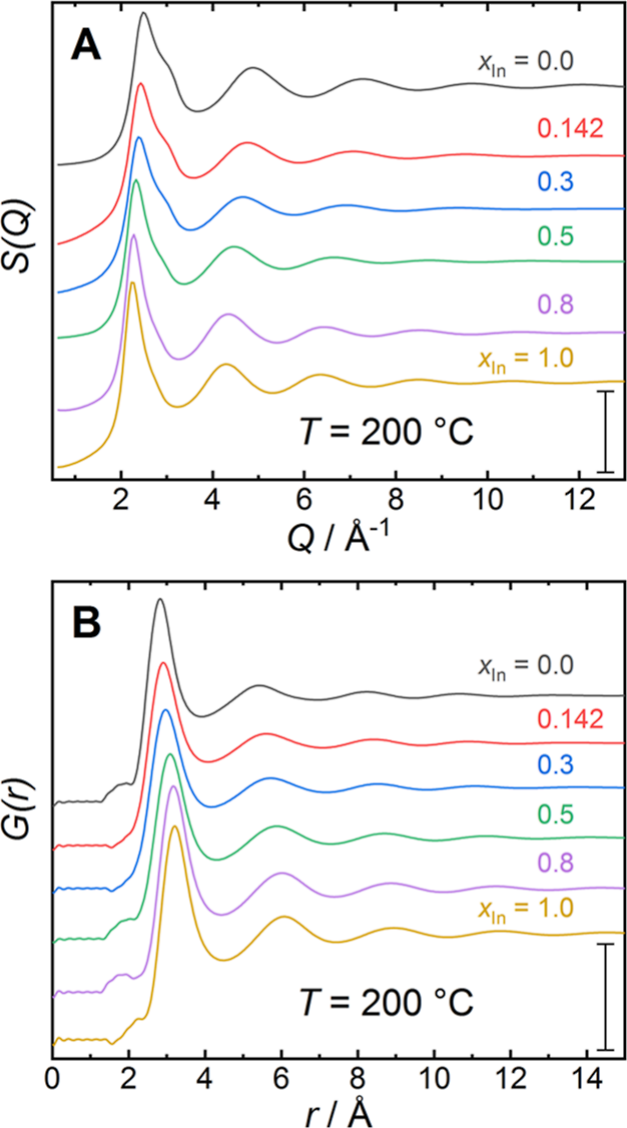
(A) Total X-ray structure factors *S*(*Q*) and (B) the derived pair distribution functions *G*(*r*) for the investigated series of liquid Ga–In alloys at *T* = 200 °C as obtained from X-ray diffraction data. Curves are shifted vertically for clarity. Scale bars in (A) and (B) represent a unit of one in *S*(*Q*) and *G*(*r*), respectively.

To investigate the local order in liquid gallium–indium alloys, synchrotron X-ray diffraction data were collected for the composition series at *T* = 150 and 200 °C. Additionally, neutron time-of-flight data were collected at *T* = 150 °C for pure gallium and the eutectic composition Ga_0.858_In_0.142_. The high neutron absorption cross section of indium precluded neutron diffraction studies at higher indium content.^50^ At 200 °C, the total scattering structure factor *S*(*Q*) for the series of alloys (Figure 2A) displays a broad first peak, characteristic for liquid alloys with a peak maximum located in the range *Q*_1_ = 2.6–2.8 Å^–1^, followed by rapidly decaying oscillations at higher *Q* values. Above *Q* = 12 Å^–1^, the structure factor becomes featureless. For the intermediate compositions *x*_In_ = 0.3 and *x*_In_ = 0.5, the oscillations in *S*(*Q*) appear to be dampened faster, indicating more structural disorder. The structure factor for pure liquid gallium (*x*_In_ = 0.0) displays a distinct high-*Q* shoulder on the first peak, which becomes less pronounced with increasing indium content. With increasing indium content, the position of the first maximum *Q*_1_ shifts gradually to lower *Q* as gallium atoms are substituted by the larger indium atoms, increasing the average nearest neighbor distance between atoms (Figure 1D). The values for *Q*_1_ at 150 and 200 °C differ only at high indium content.

The total pair distribution functions *G*(*r*) for the series of liquid Ga–In alloys at *T* = 200 °C (Figure 2B) were obtained by Fourier transform of the respective structure factors. Below *r* = 2.5 Å, *G*(*r*) shows irregular oscillations around zero due to Fourier truncation artifacts. The first peak in *G*(*r*) corresponds to the average interatomic distance in the first coordination shell and the position of the peak maximum *r*_1_ increases from *r*_1_ = 2.82 Å for *x*_In_ = 0.0 to *r*_1_ = 3.20 Å for *x*_In_ = 1.0 (Figure 1E). These values follow the metallic radii of gallium (1.4 Å) and indium (1.7 Å).^51^ At low indium concentration, the values for *r*_1_ increase linearly with In content but flatten out above *x*_In_ = 0.5.

The position of the first diffraction peak scaled by the mean atomic diameter *Q*_1_*r*_1_ has been used as a classifier of order in amorphous solids and liquids.^52^ Topological order such as in hard-sphere liquids originates from the closest approach of neighboring atoms due to the short-range repulsive potential and yields a strong peak in *G*(*r*) with *r*_1_ close to the mean atomic diameter and we find values of *Q*_1_*r*_1_ ≈ 5π/2 ≈ 7.5. Materials with additional chemical order due to Coulomb repulsion or directional bonding typically display values for *Q*_1_*r*_1_ around 4.5 or 2.5, respectively.^53^ For the investigated liquid Ga_1–*x*_In_*x*_ alloys, *Q*_1_*r*_1_ lies in the range 6.9–7.4 indicating that topological order due to size effects is prevalent (cf. Figure S2).

The average coordination number CN_avg_ in the first shell was obtained by integration of the radial distribution function *T*(*r*) = 4π ρ_0_*r*^2^*G*(*r*) up to the first minimum in *T*(*r*), where ρ_0_ is the number density. At *T* = 200 °C, a coordination number of CN_avg_ = 10.1(2) was obtained for pure gallium (*x*_In_ = 0.0). Initially, CN_avg_ remains nearly unchanged at 10.3(2) for the eutectic composition (*x*_In_ = 0.142) and then shows a strong increase to CN_avg_ = 10.9(1) for *x*_In_ = 0.5 (cf. Figure 1F). In the range *x*_In_ = 0.5–1.0, the CN_avg_ increases slowly to the maximum of CN_avg_ = 11.3(2) for pure indium. Data for CN_avg_ at *T* = 150 °C show a similar trend with a more pronounced step, increasing from 10.6(1) for liquid gallium to 11.9(2) for *x*_In_ = 0.8.

A similar trend of *Q*_1_(*x*_In_), *r*_1_(*x*_In_), and CN_avg_(*x*_In_) was also reported by Gebhardt^28^ and Vahvaselkä,^54^ who observed the change in slope around 50 at. % In and 30 at. % In, respectively. Gebhardt interpreted this observation within a micro-inhomogeneous segregation model, where two preferred local environments are present in the liquid.^28^ We used the EPSR method to create an atomistic model reproducing the experimental diffraction data of the liquid alloys and extract the partial pair distribution functions. The EPSR methodology is a Monte-Carlo simulation method where experimental diffraction data are used in combination with prior chemical information (density, pair potentials) to refine an atomistic model that describes the diffraction data.^55,56^ To improve the description of the diffraction data, empirical potentials are employed which correct the initial Lennard-Jones pair potentials to better describe the experiment. Neutron time-of-flight diffraction data were collected on the GEM diffractometer (RAL-ISIS, UK) to complement the X-ray diffraction data and examine the short-range order in liquid gallium and the liquid gallium-indium eutectic alloy (*x*_In_ = 0.142) in more detail. For the binary alloy Ga_0.858_In_0.142_, the additional scattering contrast yields more detailed information on the partial pair distribution functions *G*_ij_(*r*) and the dominant interactions in this liquid alloy system.

**Figure 3:**
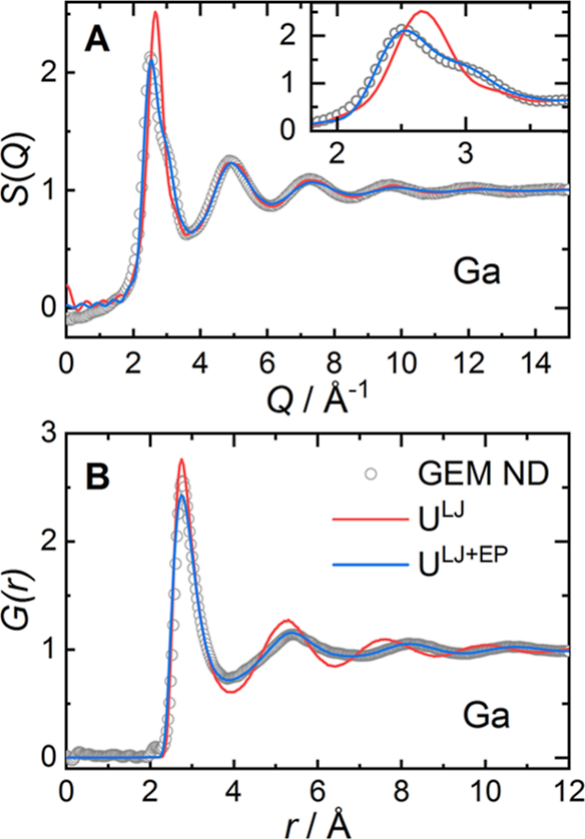
(A) Total structure factor *S*(*Q*) and (B) corresponding pair distribution function *G*(*r*) for liquid gallium at *T* = 150 °C obtained from neutron diffraction data on the GEM beamline (open circles). The simulated *S*(*Q*) and *G*(*r*) using only the Lennard-Jones potential *U*^LJ^ and using also the empirical potential *U*^LJ+EP^ are given as solid lines. Figure legend in panel B.

**Figure 4:**
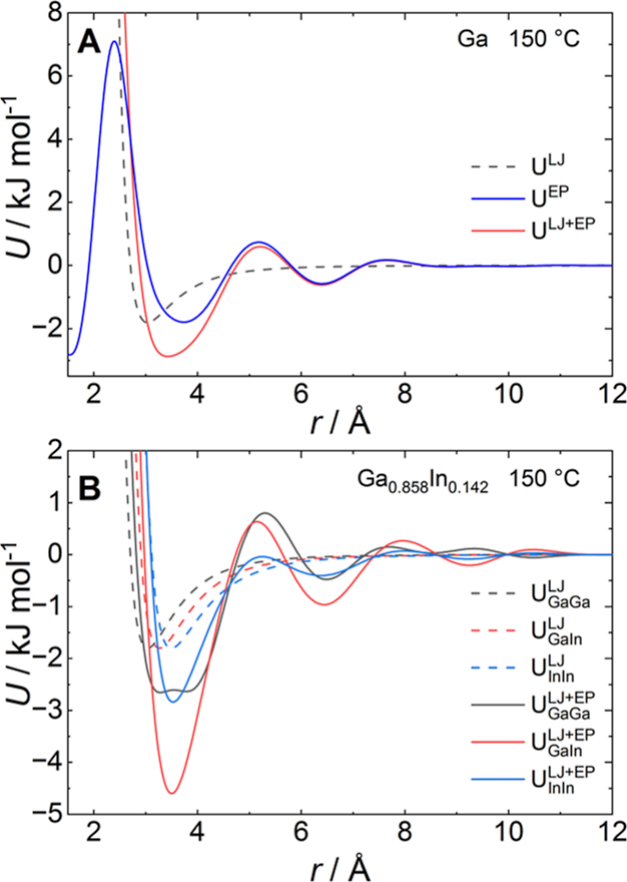
(A) Lennard-Jones potential *U*^LJ^, final empirical potential *U*^EP^, and resulting total potential *U*^LJ+EP^ for liquid gallium at *T* = 150 °C. (B) Final pair potentials for the EPSR model of the liquid eutectic Ga_0.858_In_0.142_ alloy at 150 °C. Lennard-Jones potentials *U*^LJ^ (dashed lines) and final total potentials *U*^LJ+EP^ (solid lines).

A simulation box containing 1000 gallium atoms was used to model liquid gallium at 150 °C. The converged structural model using an interatomic Lennard-Jones 12–6 potential (σ = 2.7 Å, ε = 1.8 kJ mol^–1^) was able to reproduce the general trend of *S*(*Q*) and the corresponding pair distribution function *G*(*r*) for pure gallium (Figure 3). However, this model could not reproduce the high-*Q* shoulder on the first diffraction peak in *S*(*Q*). The first coordination shell in *G*(*r*) appears well described but at larger distances the discrepancies grow. To improve the agreement between experimental and simulated structure factors, an empirical potential *U*^EP^ was introduced additionally to the initial Lennard-Jones potential *U*^LJ^ (details of the procedure are given in ref (55)(56),). Using the converged total potential *U*^LJ+EP^ improved the description of the first diffraction peak and the experimental pair distribution function. The refined empirical potential (Figure 4A) has a first minimum at *r* = 3.72 Å, followed by a dampened oscillatory behavior with minima at *r* = 6.39 Å (well depth ε = −0.57 kJ mol^–1^) and *r* = 8.85 Å (ε = −0.03 kJ mol^–1^). The total potential *U*^LJ+EP^ displays a strongly broadened minimum centered on *r* = 3.42 Å (ε = −2.87 kJ mol^–1^) and a second minimum of the pair potential located approximately 1 Å beyond the second maximum in *G*(*r*) at *r* = 5.4. The modified potential *U*^LJ+EP^ reduces the coordination number in the first shell from 11.7(1) to 10.6(1) but yields an improved description of the second and third coordination shells which are spread out to larger distances (Figure 3B). The structure model using the converged empirical potential can reproduce the characteristic shoulder on the first diffraction peak and suggests that the corresponding structural rearrangements occur not in the first but in the second and third coordination shells, similar to the simulation results of Tsai and co-workers.^9^

Here, a discussion of the refined empirical potential *U*(*r*)^EP^ with regard to the postulated interatomic pair potentials due to Friedel-type charge density oscillations is appropriate. Friedel oscillations are dampened oscillations in the interaction potential of liquid metals *U*(*r*) which originate from a discontinuity in the dielectric constant ε(*k*) of the electron gas *k* = 2 *k*_F_, where *k*_F_ is the Fermi momentum (for liquid gallium *k*_F_ ≈ 1.65 Å^–1^).^57,58^ In real space, this results in oscillations in the charge density and the pair potential with periodicity 2 *k*_F_.^59^ The radial spacing of the minima in the converged potential for gallium *U*(r)^LJ+EP^ is about 1.3 Å and does not coincide with the predicted wavelength of Friedel oscillations of λ_F_ = 2π/2 *k*_F_ = 1.92 Å in the long-range limit.^59^

Another point to consider is that while the use of the refined empirical potential led to a vastly improved description of the experimental data by the obtained atomistic model, the well depth of the second minimum in the interatomic potential *U*^LJ+EP^ is only −0.57 kJ mol^–1^, i.e., about 20% of the first minimum and only about 16% of the estimated thermal energy *k*_B_*T* at *T* = 150 °C.^60^ Most likely, the influence of the empirical potential lies therefore less with the second and third minima, but with the steep and pronounced maximum in *U*(*r*)^LJ+EP^ at *r* ≈ 5.2 Å, which pushes the second coordination shell outwards beyond the hard-sphere result and leads to the medium-range order responsible for the high-Q shoulder on the first diffraction peak. The static structure factor *S*(*Q*) for liquid indium at 200 °C (Figure S4) does not display a high-*Q* shoulder on the first diffraction peak (cf. ref (54)), but is well described by the Percus–Yevick structure factor for a hard-sphere liquid with radius σ = 1.476 Å and packing fraction φ = 0.42.^61−63^

**Figure 5:**
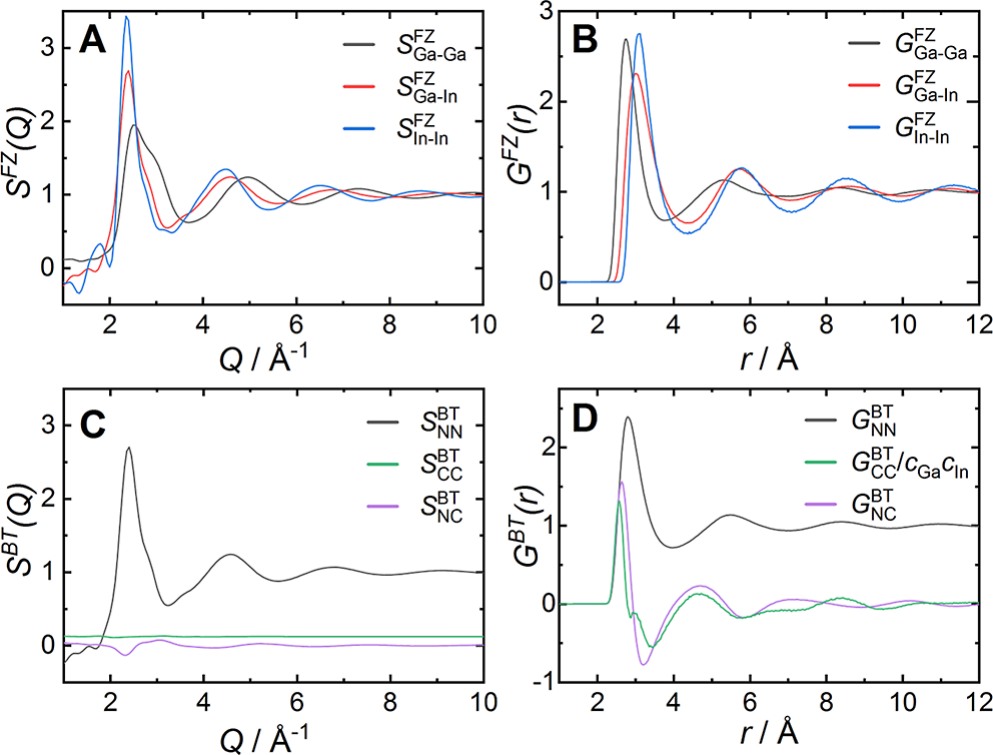
Partial structure factors and corresponding partial pair distribution functions obtained from the final EPSR model of Ga_0.858_In_0.142_ at *T* = 150 °C. Panels A, B: Faber–Ziman structure factors *S*(*Q*)^FZ^ and pair distribution functions *G*(*r*)^FZ^. Panels C, D: Bhatia-Thornton structure factors *S*(*Q*)^BT^ and pair distribution functions *G*(*r*)^BT^. *G*_CC_^BT^ is normalized by *c*_Ga_*c*_In_ for better visibility.

For one-component systems, such as pure gallium, a single diffraction contrast suffices to obtain the complete pair distribution function. For the two-component system Ga_0.858_In_0.142_, three data sets of dissimilar diffraction contrast are necessary to completely determine the three partial structure factors *S*_*ij*_(*Q*) (*i*,*j* = Ga, In) and corresponding partial pair distribution functions *G*_*ij*_(*r*). The atomistic model for the eutectic Ga_0.858_In_0.142_ was refined against both, X-ray synchrotron and neutron diffraction data. The introduction of physical constraints in the EPSR model alleviates the lack of a third diffraction contrast. The model using only Lennard-Jones potentials (σ_Ga_ = 2.70 Å, ε_Ga_ = 1.80 kJ mol^–1^, σ_In_ = 3.11 Å, ε_In_ = 1.80 kJ mol^–1^) shows approximate agreement with the experimental data. The amplitude of the empirical potential *U*^EP^ was then increased in small steps from 0 to 4 kJ mol^–1^ until no further improvement in the fit between experimental and simulated diffraction data was achieved (Figure S6). As for liquid gallium, the refined interatomic potentials *U*(*r*)^LJ+EP^ show a deep first minimum for all atom pairs, followed by oscillatory decay at larger distances (Figure 4B). The interaction between gallium and indium *U*_GaIn(*r*)_^LJ+EP^ has a particularly deep first minimum, while the gallium–gallium interaction *U*_GaGa(*r*)_^LJ+EP^ shows a strongly broadened minimum. From the atomistic model, the Faber–Ziman partial pair distribution functions *G*_*ij*_^FZ^ and their respective Fourier transforms *S*_*ij*_^FZ^ (Figure 5) were calculated. Examination of the Faber–Ziman structure factors shows that the first peak of the gallium–gallium partial structure factor *S*_GaGa(*Q*)_^FZ^ has a clear double peak structure (Figure 5A) and is responsible for the high-*Q* shoulder in the total structure factor *S*(*Q*). On the contrary, *S*_InIn(*Q*)_^FZ^ is close to a hard sphere-like structure factor. The corresponding Faber–Ziman pair distribution functions *G*_GaGa(*r*)_^FZ^, *G*_GaIn(*r*)_^FZ^*G*_InIn(*r*)_^FZ^ show sharp peaks at *r* = 2.76 Å, *r* = 3.00 Å and *r* = 3.09 Å, respectively, corresponding to the distances between atom pairs within the first coordination shell. Both *S*_GaIn(*Q*)_^FZ^ and *G*_GaIn(*r*)_^FZ^ resemble closely the indium–indium partial contributions, while the gallium–gallium contributions appear dissimilar.

The derived Bhatia–Thornton structure factors *S*_NN(*Q*)_^BT^, *S*_NC(*Q*)_^BT^, *S*_CC(*Q*)_^BT^ give the number–number, number–concentration and concentration–concentration correlations in the eutectic Ga_0.858_In_0.142_ alloy, respectively (Figure 5C).^64,65^*S*_CC(*Q*)_^BT^ shows only small oscillations around the high-*Q* limit of *c*_Ga_*c*_In_ = 0.122, indicating negligible chemical order in liquid Ga_0.858_In_0.142_. Similarly, *S*_NC(*Q*)_^BT^ shows only small deviations from zero. Hence, *S*_NN(*Q*)_^BT^ is the main contribution to the total *S*(*Q*) and topological short-range order is prominent in liquid Ga_0.858_In_0.142_. Also in real space, *G*_NN(*r*)_^BT^ is the main contribution to the total pair distribution function *G*(*r*) and closely follows the overall pair distribution function. The functions *G*_NN(*r*)_^BT^ describing the chemical order and *G*_NC(*r*)_^BT^ describing the correlation between density and concentration fluctuations show rapid small oscillations, with an approximately doubled frequency compared to *G*_NN(*r*)_^BT^. These small rapid oscillations can be ascribed to size effects and are typically present for solutions containing atoms of significantly different diameters.

Integration of the partial radial distribution functions *T*_*i*_^*j*^(*r*) = 4π *c*_*j*_ ρ_0_*r*^2^*G*_ij(*r*)_^FZ^ up to the first minimum yields the partial coordination numbers CN_*i*_^*j*^ for atoms of type *j* around atoms of type *i* in the first coordination shell. The values for liquid Ga_0.858_In_0.142_ at *T* = 150 °C are CN_Ga_^Ga^ = 8.5(1), CN_Ga_^In^ = 2.1(2), CN_In_^Ga^ = 12.5(1) and CN_In_^In^ = 2.0(4). The corresponding Warren-Cowley short-range order parameter for the first coordination shell α_WC_ = −0.04 is close to the value for a statistical distribution (α_WC_ = 0).^66^ The absence of strong interactions, e.g., due to charge transfer between Ga and In, is in line with constant atomic volumes across the composition series, as evidenced by the negligible molar excess volumes (cf. Figure 1C).

## Conclusions

Neutron diffraction data and EPSR simulation results on liquid gallium at 150 °C have provided experimental support for the hypothesis that the high-Q shoulder observed on the first diffraction peak does not originate from gallium dimers in the melt, but rather from structural rearrangements in the second coordination shell. The empirical potential *U*^EP^ refined for Ga from EPSR simulations qualitatively resembles the results expected for an ordering induced by Friedel oscillations;^9^ however, quantitatively, the oscillation period does not match well with theoretical predictions.

Analysis of short-range order in the eutectic alloy Ga_0.858_In_0.142_ did not indicate a strong preference for hetero- or homo-atomic interactions, as evidenced by inconspicuous behavior of *S*_CC(*Q*)_^BT^ and a near-zero Warren-Cowley order parameter. The absence of strong chemical interactions, such as charge transfer, is also evidenced by the constant atomic volumes across the composition range.

EPSR analysis at the eutectic composition suggests a particularly deep minimum in the pair potential *U*_GaIn(*r*)_^LJ+EP^ which could originate from a maximum in packing density as observed for unequal hard spheres around 20% fraction of the larger spheres.^67^ An inflection point in the liquidus curve around the equiatomic composition had also been observed in the Ag–Sn system and was suggested as a composition with particularly difficult packing of unequal atoms. Similarly to the results of Gebhardt, the presence of two types of incompatible coordination environments or cluster types was postulated.^46,47^

Extrapolating from the detailed analysis of the eutectic alloy to the In-rich compositions, preferred interactions between atom pairs may be excluded as cause for the reported positive enthalpy of mixing Δ*H*_M_ in this system. In line with the symmetric curve of Δ*H*_M_(*x*), also valence effects can be excluded for alloys of these isoelectronic elements. While the measured partial atomic volumes are constant across the composition series, the volumetric density and therefore also density of the electron gas in the liquid alloys is increased relative to the elemental densities. Similar to the binary alkali metal alloys, the observed positive mixing enthalpy in Ga–In alloys can therefore be related to electronic effects, as the charge density is modified from the equilibrium values of the elemental end members.^68,69^

The results collected from volumetric density and diffraction data across the composition series, as well as EPSR simulations for liquid Ga and liquid Ga_0.858_In_0.142_, corroborate that the structure of liquid gallium has anomalous features compared to simple hard-sphere liquids, which manifest in a redistribution of Ga atoms from the first to the second coordination sphere, leading to an observable high-*Q* shoulder in diffraction data. Determining the reason for this anomalous behavior will require additional studies. Binary Ga–In alloys, on the other hand, show no indication of preferred atomic interactions in trends of molar excess volumes or analysis of chemical short-range order. Mixing with indium appears to “dilute” the anomalous properties of liquid Ga and especially on the indium-rich side the effect of Ga on the diffraction data (high-Q shoulder) vanishes.

## Supporting Information Available

Additional figures on density measurements, diffraction data analysis, and interatomic potentials; table of measured density values (PDF)
