# Supplementary material for: Local Order in Liquid Gallium–Indium Alloys
Source: J Phys Chem C Nanomater Interfaces. 2023 Aug 9;127(33):16687–94. doi: 10.1021/acs.jpcc.3c03857 (PMC10461300; doi:10.1021/acs.jpcc.3c03857)
Supplement: Supplementary file 1 — jp3c03857_si_001.pdf [file jp3c03857_si_001.pdf]

Supporting information for the manuscript: Local Order in Liquid Gallium-Indium Alloys

Alfred Amon[*†#], *Philip A. Chater,*[‡] *Gavin Vaughan, Rachael Smith,*[†] *Christoph G. Salzmann*[†]

AUTHOR ADDRESS

† Department of Chemistry, University College London, 20 Gordon Street, WC1H 0AJ London, United Kingdom. ‡ Diamond Light Source Ltd., Harwell Science and Innovation Campus, Didcot OX11 0DE, U.K.   European Synchrotron Radiation Facility, BP-220, F-38043, Grenoble Cedex 9, France. # Material Science Division, Lawrence Livermore National Laboratory, 94550 Livermore, CA, USA.

FIGURES

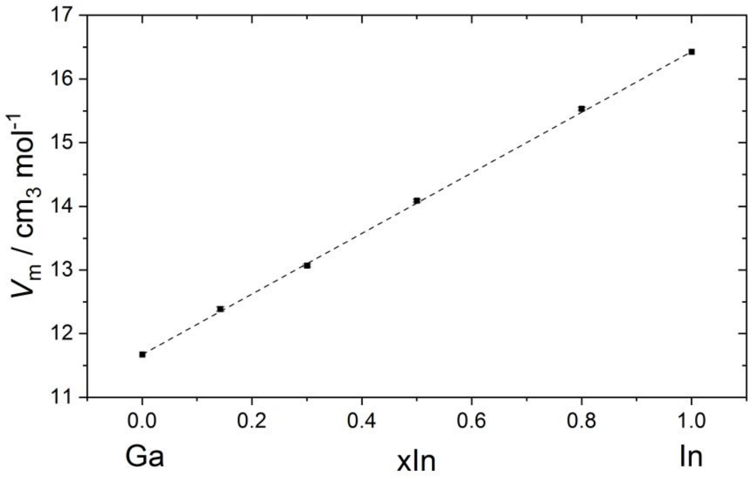

Figure S1: Experimental molar volumes of liquid Ga-In alloys at T = 200 ˚C. Error bars indicating one e.s.d. are within the symbols. The dashed line represents Zen’s law, or ideal solution behavior.

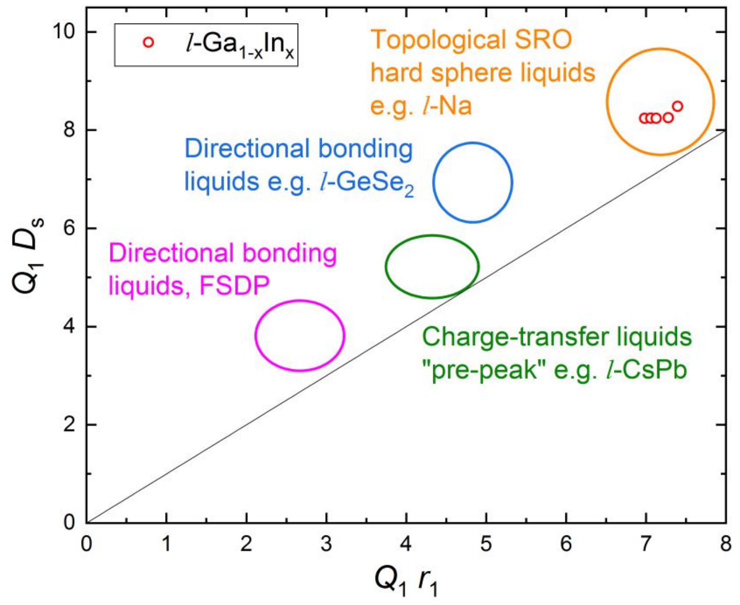

Figure S2: The product $Q_1 r_1$ for liquid Ga-In alloys at T = 200 ˚C (small red circles), scaled by $Q_1 D_s$, where $D_s = \left(\frac{6}{\pi\rho_0}\right)^{1/3}$ gives the mean atomic spacing. Large empty circles indicate typical ranges for amorphous materials with different types of dominant short-range order [1].

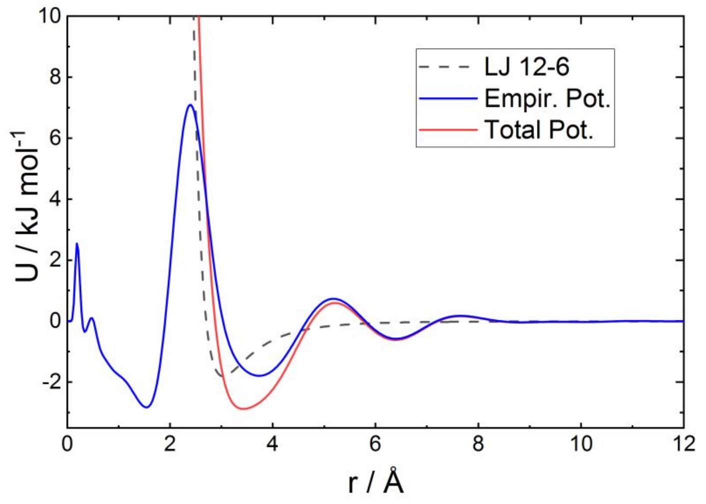

Figure S3: Lennard-Jones potential U^{LJ} (dashed line), final empirical potential U^{EP} (blue solid line) and resulting total potential U^{LJ+EP} (red solid line) for liquid gallium at T = 150 ˚C.

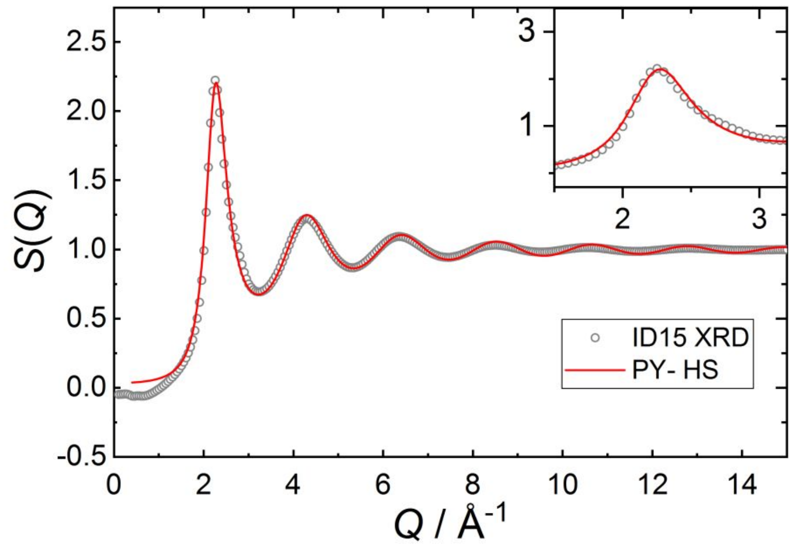

Figure S4: Experimental structure factor S(Q) for liquid indium at 200 ˚C (open circles) from synchrotron x-ray diffraction. The Percus-Yevick solution for the hard-sphere liquid structure factor with hard-sphere radius σ = 1.476 Å and packing fraction φ = 0.42 is given as solid line [2–4].

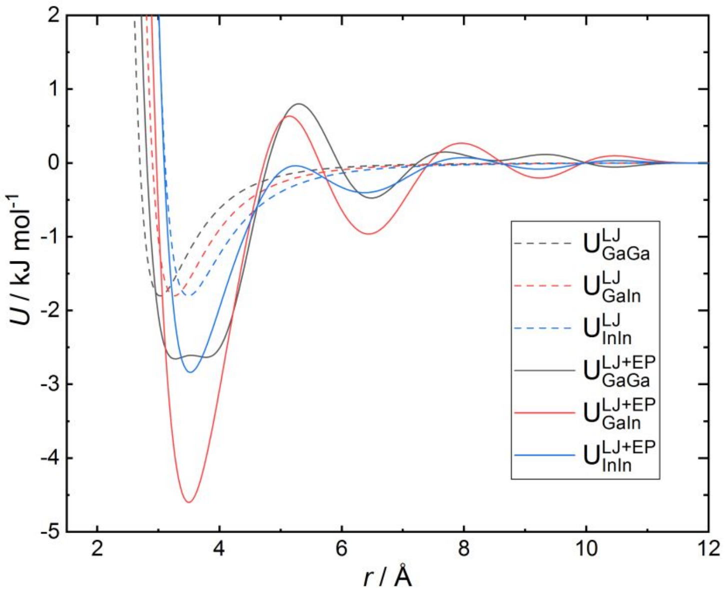

Figure S5: Final potentials for the EPSR model of the liquid eutectic $Ga_{0.858}In_{0.142}$ alloy at 150 ˚C. Lennard-Jones potentials U^{LJ} (dashed lines) and final total potentials U^{LJ+EP} (solid lines).

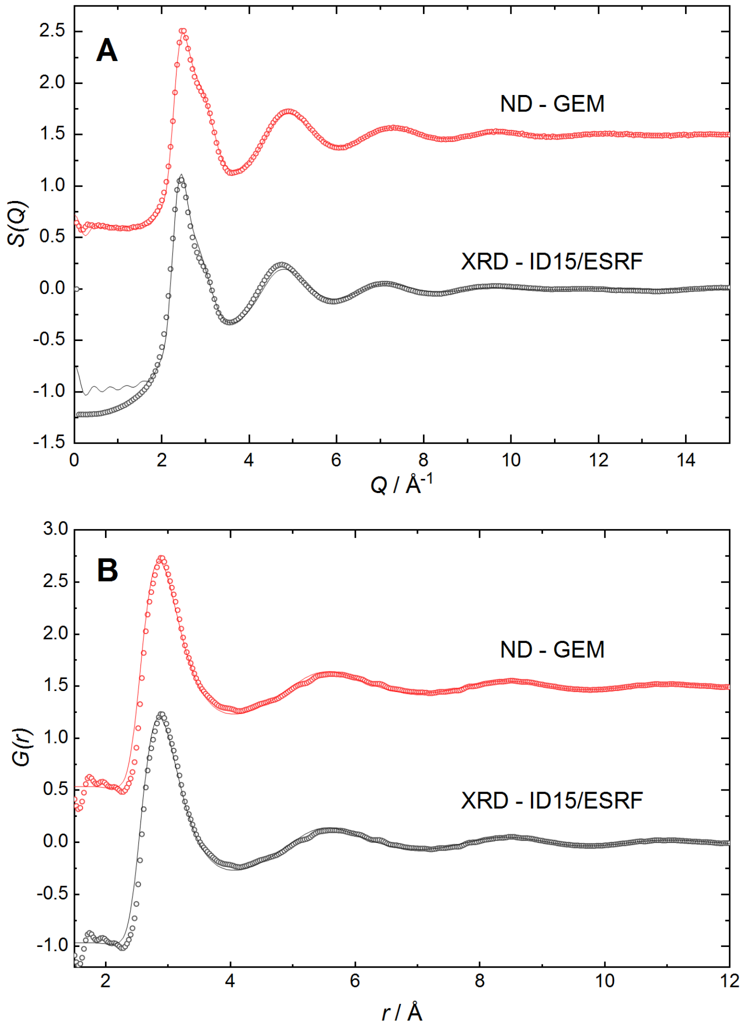

Figure S6: Total structure factors $S(Q)$ and corresponding total pair distribution functions $G(r)$ for the liquid $Ga_{0.858}In_{0.142}$ alloy from neutron diffraction (ND) and synchrotron x-ray diffraction (XRD). Experimental data are given as open circles and simulated data from the EPSR model are given as solid lines. Data are shifted vertically for visibility.

Table S1: Experimental values for density and molar volume of liquid Ga-In alloys at $T = 200$ °C.

Composition	Density / g cm^{-3}	Molar Volume / cm^3 mol^{-1}	Ideal molar volume / cm^3 mol^{-1}
Ga	5.9737(18) [5]	11.671	11.671
$Ga_{0.858}In_{0.142}$	6.15(2)	12.38(4)	12.347
$Ga_{0.7}In_{0.3}$	6.37(1)	13.07(2)	13.099
$Ga_{0.5}In_{0.5}$	6.55(1)	14.09(2)	14.050
$Ga_{0.2}In_{0.8}$	6.813(1)	15.53(2)	15.477
In	6.989 [6]	16.429	16.429

AUTHOR INFORMATION

Corresponding Author

* amon1@llnl.gov

REFERENCES FOR SUPPORTING INFORMATION

[1] M. Saboungi, W. Geertsma, D.L. Price, Ordering in Liquid Alloys, Annu. Rev. Phys. Chem. 41 (1990) 207–244. https://doi.org/10.1146/annurev.pc.41.100190.001231.

[2] J.K. Percus, Approximation Methods in Classical Statistical Mechanics, Phys. Rev. Lett. 8 (1962) 462–463. https://doi.org/10.1103/PhysRevLett.8.462.

[3] J.K. Percus, G.J. Yevick, Analysis of Classical Statistical Mechanics by Means of Collective Coordinates, Phys. Rev. 110 (1958) 1–13. https://doi.org/10.1103/PhysRev.110.1.

[4] C.G. Gray, K.E. Gubbins, Theory of Molecular Fluids: I: Fundamentals, Oxford University Press, Oxford, 1984. https://doi.org/10.1093/oso/9780198556022.001.0001.

[5] H. Köster, F. Hensel, E. Franck, Dichte, Kompressibilität und thermische Ausdehnung des flüssigen Galliums bis 600 ˚C und 2500 bar., in: 1970. https://doi.org/10.1002/BBPC.19700740113.

[6] M.J. Assael, I.J. Armyra, J. Brillo, S.V. Stankus, J. Wu, W.A. Wakeham, Reference Data for the Density and Viscosity of Liquid Cadmium, Cobalt, Gallium, Indium, Mercury, Silicon, Thallium, and Zinc, J. Phys. Chem. Ref. Data. 41 (2012) 033101. https://doi.org/10.1063/1.4729873.
